# Mutation spectrum of the *OPA1* gene in a large cohort of patients with suspected dominant optic atrophy: Identification and classification of 48 novel variants

**DOI:** 10.1371/journal.pone.0253987

**Published:** 2021-07-09

**Authors:** Nicole Weisschuh, Simone Schimpf-Linzenbold, Pascale Mazzola, Sinja Kieninger, Ting Xiao, Ulrich Kellner, Teresa Neuhann, Carina Kelbsch, Felix Tonagel, Helmut Wilhelm, Susanne Kohl, Bernd Wissinger

**Affiliations:** 1 Institute for Ophthalmic Research, Centre for Ophthalmology, University of Tübingen, Tübingen, Germany; 2 CeGaT GmbH and Praxis für Humangenetik Tübingen, Tübingen, Germany; 3 Institute of Medical Genetics and Applied Genomics, University of Tübingen, Tübingen, Germany; 4 Zentrum für seltene Netzhauterkrankungen, AugenZentrum Siegburg, MVZ Augenärztliches Diagnostik- und Therapiecentrum Siegburg GmbH, Siegburg, Germany; 5 RetinaScience, Bonn, Germany; 6 Medical Genetics Center, Munich, Germany; 7 Centre for Ophthalmology, University Eye Hospital, University of Tübingen, Tübingen, Germany; University of Ferrara: Universita degli Studi di Ferrara, ITALY

## Abstract

Autosomal dominant optic atrophy is one of the most common inherited optic neuropathies. This disease is genetically heterogeneous, but most cases are due to pathogenic variants in the *OPA1* gene: depending on the population studied, 32–90% of cases harbor pathogenic variants in this gene. The aim of this study was to provide a comprehensive overview of the entire spectrum of likely pathogenic variants in the *OPA1* gene in a large cohort of patients. Over a period of 20 years, 755 unrelated probands with a diagnosis of bilateral optic atrophy were referred to our laboratory for molecular genetic investigation. Genetic testing of the *OPA1* gene was initially performed by a combined analysis using either single-strand conformation polymorphism or denaturing high performance liquid chromatography followed by Sanger sequencing to validate aberrant bands or melting profiles. The presence of copy number variations was assessed using multiplex ligation-dependent probe amplification. Since 2012, genetic testing was based on next-generation sequencing platforms. Genetic screening of the *OPA1* gene revealed putatively pathogenic variants in 278 unrelated probands which represent 36.8% of the entire cohort. A total of 156 unique variants were identified, 78% of which can be considered *null* alleles. Variant c.2708_2711del/p.(V903Gfs*3) was found to constitute 14% of all disease-causing alleles. Special emphasis was placed on the validation of splice variants either by analyzing cDNA derived from patients´ blood samples or by heterologous splice assays using minigenes. Splicing analysis revealed different aberrant splicing events, including exon skipping, activation of exonic or intronic cryptic splice sites, and the inclusion of pseudoexons. Forty-eight variants that we identified were novel. Nine of them were classified as pathogenic, 34 as likely pathogenic and five as variant of uncertain significance. Our study adds a significant number of novel variants to the mutation spectrum of the OPA1 gene and will thereby facilitate genetic diagnostics of patients with suspected dominant optic atrophy.

## Introduction

Neuropathies of the optic nerve severely impair vision. They mainly affect visual acuity, central visual fields and color vision due to a progressive loss of retinal ganglion cells and their axons. Optic neuropathies can be divided into inherited and acquired forms. The latter mostly result from vessel occlusions, extrinsic or intrinsic lesions, optic neuritis, neurotoxic substances, nutritional deficiencies, viral infections, and mixed etiologies [1]. The two most common inherited optic neuropathies seen in clinical practice are Leberʼs hereditary optic neuropathy (LHON; MIM#535000) and dominant optic atrophy (DOA; MIM#165500) [2]. The prevalence of DOA ranges between 1 in 12,000 in Denmark due to a founder mutation and 1 in 50,000 in the rest of the world [3–5].

DOA usually presents with slowly progressive and bilateral visual impairment in the first decade of life, while a correct diagnosis is often only made in the second decade [6, 7]. The main clinical features are reduced visual acuity, central visual field loss and color vision disturbances mainly in the tritan axis [8, 9]. DOA severity is highly variable: visual acuity can range from 20/20 to light perception with 40% of patients having a visual acuity better than 20/60 [10]. On the other hand, extra-ocular manifestations in DOA, often referred to as DOAplus phenotypes, are reported in up to 20% of DOA cases and include sensorineural hearing loss, progressive external ophthalmoplegia, peripheral neuropathy and ataxia [11–15].

DOA is genetically heterogeneous. Pathogenic variants in *OPA1*, which was the first gene to be described as an underlying cause of DOA [16–23], are found in 32–90% of DOA cases, depending on the population studied, the number of genes analyzed and the platform used. Other genes associated with DOA include *WFS1* [24–27], *OPA3* [28–33], *AFG3L2* [34, 35], *SPG7* [34, 36], *DNM1L* [37], *MFN2* [38], *SSBP1* [39–41], *NR2F1* [42–45], and *ACO2* [46]. Notably, with the exception of *OPA1*, all DOA-associated genes were first identified in the context of syndromic neurodegenerative diseases, and only later shown to also cause non-syndromic DOA [47]. Despite rather comprehensive workup using next generation sequencing technologies, more than one third of the patients remain without the identification of the genetic cause of their disease [48].

The *OPA1* gene (MIM #605290) is located on chromosome 3q29 and encodes for a ubiquitously expressed dynamin-related GTPase, which is imported into mitochondria by an N-terminal import sequence and localizes to the inner membrane facing the intermembrane space [49, 50]. Together with mitofusin 1, OPA1 plays a crucial role in mitochondrial fusion and is therefore vital for the maintenance of the mitochondrial network and morphology [51, 52]. In addition, OPA1 participates in cytochrome c release and reduced OPA1 expression has been associated with a significant impairment of oxidative phosphorylation [53, 54]. The *OPA1* gene is composed of 30 coding exons distributed across more than 90 kb of genomic DNA. Alternative splicing of exons 4, 4b and 5b gives rise to eight different isoforms with open reading frames for polypeptides of 960 to 1015 amino acid residues [55].

As of April 2021, the Human Gene Mutation Database (HGMD) [56] lists more than 400 disease-causing variants in *OPA1* while the Leiden Open Variation Database database for *OPA1* (https://www.lovd.nl/OPA1) [57], which entries overlap largely but not completely with HGMD, lists 593 unique public variants. Haploinsufficiency has been proposed as the predominant pathomechanism for *OPA1* variants [18, 57]. Accordingly, the majority of disease-causing variants are predicted to give rise to truncated OPA1 polypeptides, either due to nonsense, frameshift or splice variants, the latter including deep intronic variants causing the inclusion of pseudoexons into the transcript [58, 59]. In addition, structural variants such as copy number variants (CNVs) and inversions are part of the mutation spectrum of *OPA1* [60–62]. Disease-causing variants are spread over the entire coding sequence, however, very few have been reported for the alternatively spliced exons [63].

Notably, patients who are compound heterozygous for two pathogenic *OPA1* alleles are very rare. In fact, patients with biallelic *null* alleles have never been reported, probably because such a genotype is most likely embryonic lethal, as suggested by animal models [64–66]. Most patients (~80%) with biallelic *OPA1* variants harbor a truncating variant on one allele and a (hypomorphic) missense variant on the counter allele. These patients present with Behr (MIM#210000) or Behr-like syndrome, a distinct severe neurological syndromic disease characterized by early-onset optic atrophy, spasticity, spinocerebellar ataxia, peripheral neuropathy, gastrointestinal dysmobility and sometimes intellectual disability [15, 58, 67–70]. With respect to phenotype-genotype correlations, missense variants are more likely to cause a more severe phenotype than *null* alleles [71, 72], potentially due to a dominant-negative effect caused by partially inactive OPA1 homopolymers [73]. In general, genotype-phenotype correlations in *OPA1*-associated diseases are hampered by the highly variable clinical expression observed both between and within families harboring the same variant [20]. In fact, the penetrance may vary considerably, between 43 to 88% [19, 74, 75], indicating the presence of yet undefined modifying factors that have the potential to modulate the phenotypic expression of DOA.

The aim of the present study was to provide a comprehensive overview over the entire spectrum of likely pathogenic variants in the *OPA1* gene in a large cohort of patients diagnosed with DOA that has been genetically analysed over a period of 20 years since the original identification of the *OPA1* gene in 2000 [16, 17]. Hence, many variants have already been described and published by us. In this study, we present and classify 48 as yet unpublished *OPA1* variants, which adds a considerable number of novel variants to the mutation spectrum of *OPA1*, thereby facilitating the genetic diagnosis in future patients.

## Methods

### Editorial policies and ethical considerations

This was a retrospective cohort study of patients with a clinical diagnosis of DOA that were recruited between July 1992 and December 2020 in several ophthalmic centers mainly in Germany, and sent to the Institute for Ophthalmic Research in Tübingen (Germany) for genetic investigation. Other referring centers are located in Italy, France, Belgium, The Netherlands, UK, Spain, and Israel. Samples from all patients and family members were recruited in accordance with the principles of the Declaration of Helsinki. All patients provided informed written consent to use their medical records and samples for research purposes. For the probands who were underage at the time of recruitment, informed written consent was obtained from the probands’ parents or guardians. Specifically, this study was approved by the institutional review board of the Ethics Committee of the University Hospital of Tübingen under the study numbers 112/2001, 598/2011BO1 and 637/2017BO1. Data were not anonymized prior analysis.

### Subjects and clinical diagnosis

Demographic data assessed in this study included age, gender and place of residence (see Table 1). Inclusion criteria were a history of gradual, bilateral loss of vision associated with the presence of central or caeco-central scotoma on visual field evaluation and symmetric temporal or diffuse optic disc pallor. Ocular coherence tomography (OCT) findings like thinning of retinal ganglion cell fibers and reduction of the peripapillary retinal nerve fiber layer were not a prerequisite for patients to be included in this study.

**Table 1:** Demographic data of study cohort.

Mean age^a^	47.4±17.5
Range years^a^	9–93
**Gender**	
Male	429
Female	311
Not known	15
**Country of residence**	
Germany	587
Italy	69
France	35
Belgium	12
UK	9
Israel	7
The Netherlands	4
Spain	2
Other	30

^a^related to birth date and not to age at recruitment

### DNA and RNA isolation

Genomic DNA was extracted from venous blood samples applying standard salting-out procedure or by using the chemagic™ MSM1 system and the chemagic™ DNA Blood 7k Kit (Chemagen, Baesweiler, Germany). For RNA isolation whole blood was collected in PAXgene blood RNA tubes and RNA was isolated using the PAXgene blood RNA Kit (Qiagen, Hilden, Germany). Alternatively, leukocytes were isolated from venous blood by Ficoll-Paque density centrifugation (Pharmacia Biotech, Freiburg, Germany) and total RNA was extracted with Trizol reagent (Life Technologies, Eggenstein, Germany).

### Mutation screening of the *OPA1* gene

Genetic analysis has changed over the years, following the implementation of novel analysis techniques. From 2000–2012, the coding region was analyzed by a combined approach using either single-strand conformation polymorphism (SSCP) or denaturing high performance liquid chromatography (DHPLC) followed by Sanger sequencing to validate aberrant bands or melting profiles [18]. Sanger sequencing of all coding exons was performed in individual cases. For cDNA analysis, RNA obtained from blood samples was reverse transcribed by using the SuperScript First-Strand Synthesis System for RT-PCR (Invitrogen, Carlsbad, CA, USA) [76]. Screening for large-scale rearrangements was performed using multiplex ligation-dependent probe amplification (MLPA) [60]. Since 2012, patients’ DNAs were subjected to next-generation sequencing (NGS), either using a panel-based approach or whole genome sequencing [62, 77].

### Variant nomenclature

Genomic coordinates given in this manuscript are based on the GRCh37 genome assembly. Mutation nomenclature is based on GenBank accession NM_015560.2 with nucleotide one being the first nucleotide of the translation initiation codon ATG. This isoform lacks the alternative exons 4b and 5b. Mutation nomenclature was validated using the Mutalyzer name checker tool (https://mutalyzer.nl/name-checker). All variants were cross-checked with a literature search performed on February 1st 2021, HGMD [56], and the Leiden Open Variation Database database for *OPA1* (https://www.lovd.nl/OPA1) [57]. Only variants not listed in HGMD, LOVD or published in a scientific journal searchable on Pubmed (https://pubmed.ncbi.nlm.nih.gov/) were referred to as “novel”. All novel variants have been submitted to the "Global Variome shared LOVD" (databases.lovd.nl/shared/references/ DOI: 10.1371/journal.pone.0253987).

### *In silico* analyses

Novel variants identified in this study were evaluated with different web-based databases and tools. Allele frequencies were retrieved from the Genome Aggregation Database (gnomAD v2.1.1) [78]. The pathogenicity of missense variants was assessed with MutationTaster, which integrates information from different biomedical databases and uses established analysis tools including evolutionary conservation, splice-site changes, loss of protein features and changes that might affect the amount of mRNA [79]. In addition, the online prediction tools PolyPhen-2 (http://genetics.bwh.harvard.edu/pph2/), and PROVEAN and SIFT (http://provean.jcvi.org/genome_submit_2.php), were used to predict the impact of missense variants. Protein sequence alignment of *Homo sapiens* OPA1 against its orthologues from twelve other species was performed using Clustal Omega [80]. Non-canonical splice site variants were evaluated with Alamut Visual v.2.14.0 (Interactive Biosoftware, Rouen, France, www.interactive-biosoftware.com/alamut-visual/), which allows a simultaneous analysis with the programs NNSPLICE [81], MaxEntScan [82], SpliceSiteFinder-like [83], and GeneSplicer [84]. All tools were used according to the guidelines for the use of prediction tools [85].

### Variant classification

Classification of novel variants identified in this study adhered to the guidelines published by the American College of Medical Genetics and Genomics and the Association for Molecular Pathology (ACMG/AMP) [86].

### Minigene splice assays

Minigene splice assays were performed as described previously [76, 87]. Briefly, genomic segments encompassing the variant of interest along with flanking sequences were amplified from patient genomic DNA using a proofreading polymerase and cloned into the pSPL3 minigene plasmid vector [88, 89]. Specifically, cloned genomic segments were 1092 bp for the analysis of variant c.2014-10A>G (GrCh37/hg19 3: 193,374,384–193,375,475; corresponding to exon 21 and flanking intronic sequences), and 1634 bp for the analysis of variant c.2356-8T>G (GrCh37/hg19 3: 193,379,822–193,381,455; corresponding to exon 24 and flanking intronic sequences). Following cloning, the resulting constructs in their wildtype and mutant version were used to transfect HEK293T/17 cells (ATCC® CRL-11268™), which were then analyzed with respect to splicing of minigene-derived transcripts by reverse transcription polymerase chain reaction (RT-PCR) using vector-specific primers (F: 5´-TGGACAACCTCAAAGGCACC-3´and R: 5´-AGTGAATTGGTCGAATGGATC-3´).

### Quantification of RT-PCR products

Four hundred ng of total RNA isolated from blood samples was used for cDNA synthesis using random hexamers and the Maxima H Minus Reverse Transcriptase according to the manufacturer’s protocol (Thermo Fisher Scientific, Carlsbad, USA). For the analysis of variants c.1065+6T>C and c.1212+4del, reverse transcription polymerase chain reaction (RT-PCR) was performed using 2 μl cDNA, a forward primer located in exon 7 (5´- TGGAACGATTAGAAAAGGAGAACAAAG-3´), a 5′ FAM (6-carboxyfluorescein) labeled reverse primer located in exon 14 (5´- CCATGAGGGTCCATTTGACT-3´), and standard PCR conditions (35 cycles). For the analysis of variant c.1516+3A>G, a forward primer located in exon 11 (5´- GAACTTCGAATGAGGAAAAATGTGA-3´) and a 5′ FAM (6-carboxyfluorescein) labeled reverse primer located in exon 17 (5´- TGTTGTTCAACAGACTCTCGTACCAT-3´) was used.

FAM-labeled RT-PCR products were mixed with 0.5 μl of GeneScan ROX500 size standard (Life Technologies, Darmstadt, Germany) and 8.5 μl of Hi-Di Formamide (Life Technologies) in a total volume of 10 μl. Mixes were separated by capillary electrophoresis on an ABI 3130XL Genetic Analyzer instrument (Life Technologies). The area-under-the-curve (AUC) was calculated with GeneMapper 5 (Life Technologies) software. Ratios of RT-PCR products were determined as the AUC for individual peaks divided by the sum of AUC of all peaks.

## Results

**Fig 1:**
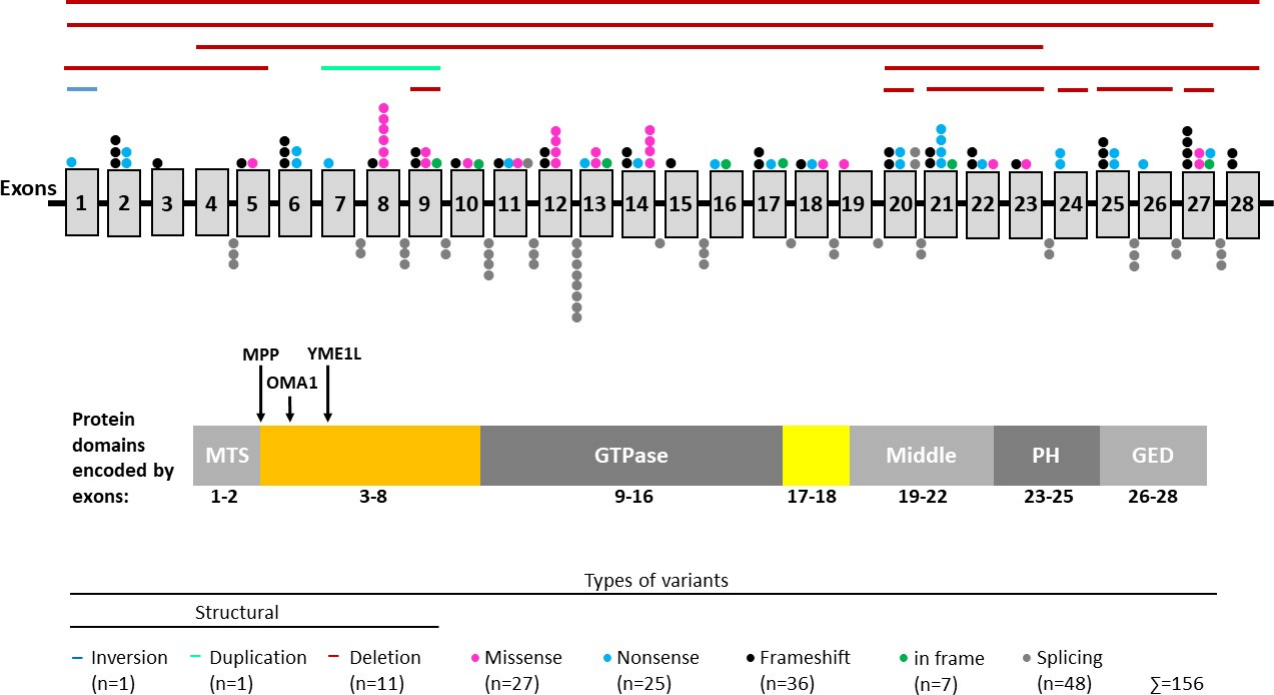
Distribution of *OPA1* variants. Shown is the isoform that lacks the alternative exons 4b and 5b (NM_015560.2). Exons are represented by grey vertical boxes. Note that exons and intervening intronic sequence (represented by black horizontal line) are not drawn to scale. Each distinct variant observed in our cohort is represented by a single distinct color coded dot above the respective exon or below the respective intron. Structural variants are indicated by horizontal lines above the exons. Shown below the gene structure is the protein with its most important domains including a GTPase domain, a middle domain that is involved in oligomerization, a pleckstrin homology (PH) domain and a GTPase effector domain (GED). The peptide encoded by exons 17–18 (shown in yellow) forms a long helix that connects the GTPase domain and the middle domain. Exons 1 and 2 encode a mitochondrial targeting sequence (MTS) which is cleaved by the mitochondrial processing peptidase (MPP). The N-terminal region encoded by exons 3–8 (shown in orange) does not include specific domains but comprises mitochondrial proteolytic cleavage sites for the mitochondrial processing peptidases OMA1 and YME1L. Protein structure was adapted from Li et al., 2019 [109].

Our cohort of clinically diagnosed DOA patients comprises 755 index patients. Demographic data are shown in Table 1. Genetic screening of the *OPA1* gene identified putatively pathogenic variants in 278 unrelated individuals. Known and novel variants are presented in Tables 2 and 3, respectively, and the distribution of variants along the gene is shown in Fig 1.

**Table 2:** Known variants identified in this study.

NM_015560.2	NP_056375.2^a^	Exon/Intron	HGMD^b^
c.6G>A	p.(W2*)	Exon 1	CM012163
c.112C>T	p.(R38*)	Exon 2	CM024785
c.154C>T	p.(R52*)	Exon 2	CM076368
c.557-668G>A	p.(S187Afs*28)/p.(S187Afs*29)	Intron 4	CS147231
c.557-672G>A	p.(G186Afs*9)	Intron 4	CS147232
c.629C>A	p.(S210*)	Exon 6	CM012164
c.631_634del	p.(D211Kfs*16)	Exon 6	CD072458
c.635_636del	p.(K212Rfs*4)	Exon 6	CD012268
c.639_640del	p.(K214Nfs*2)	Exon 6	CD104762
c.655C>T	p.(Q219*)	Exon 6	CM111745
c.703C>T	p.(R235*)	Exon 7	CM136994
c.784-1G>A	p.(K262_R290del)	Intron 7	CS080724
c.784-2A>G	p.(?)	Intron 7	CS1410777
c.808G>A	p.(E270K)	Exon 8	CM012165
c.815T>C	p.(L272P)	Exon 8	CM031310
c.818A>C	p.(D273A)	Exon 8	CM012166
c.869G>A	p.(R290Q)	Exon 8	CM002636
c.868C>T	p.(R290W)	Exon 8	CM012167
c.870+1del	p.(?)	Intron 8	LOVD-ID:OPA1_000475
c.870+1G>A	p.(?)	Intron 8	CS1410779
c.870+5G>A	p.(?)	Intron 8	CS012215
c.895G>C	p.(A299P)	Exon 9	CM080471
c.932del	p.(A311Vfs*11)	Exon 9	CD012270
c.937_938delinsGA	p.(I313E)	Exon 9	CP015804
c.984+2T>A	p.(V291_K328del)	Intron 9	CS080725
c.984+3A>T	p.(?)	Intron 9	CS024779
c.992T>C	p.(L331P)	Exon 10	CM131132
c.1065+1G>A	p.(V329_D355del)	Intron 10	CS080718
c.1065+3A>C	p.(?)	Intron 10	CS012216
c.1065+5G>A	p.(?)	Intron 10	CS1410781
c.1072_1093del	p.(A358Ffs*3)	Exon 11	PMID:11440988
c.1096C>T	p.(R366*)	Exon 11	CM002638
c.1126A>G	p.(T376A)	Exon 11	CM014008
c.1140G>A	p.(L356_E380del)	Exon 11	CS061311
c.1140+1dup	p.(L356_E380del)	Intron 11	CI080971
c.1140+5G>C	p.(L356_E380del)	Intron 11	CS080719
c.1410_1443+4del	p.(V452_R481del)	Exon 14/Intron 14	CG084686
c.1146A>G	p.(I382M)	Exon 12	CM080464
c.1153_1154del	p.(N385Cfs*13)	Exon 12	CD012271
c.1198C>T	p.(P400S)	Exon 12	CM080462
c.1199C>T	p.(P400L)	Exon 12	CM080465
c.1212_1212+4del	p.(?)	Intron 12	CD056198
c.1212+1G>A	p.(T381_N404del)	Intron 12	CS014017
c.1212+1G>T	p.(?)	Intron 12	CS013627
c.1212+5G>C	p.(T381_N404del)	Intron 12	CS080720
c.1213-2A>G	p.(T405fs*9)	Intron 12	CS094201
c.1279C>T	p.(Q427*)	Exon 13	CM080466
c.1296_1298del	p.(I433del)	Exon 13	CD002706
c.1301T>C	p.(L434P)	Exon 13	CM1814099
c.1313A>G	p.(D438G)	Exon 14	CM066157
c.1313A>T	p.(D438V)	Exon 14	CM012169
c.1334G>A	p.(R445H)	Exon 14	CM030379
c.1346dup	p.(D450Rfs*38)	Exon 14	CI080972
c.1354del	p.(V452Sfs*15)	Exon 14	CD002707
c.1402A>G	p.(K468E)	Exon 14	CM012170
c.1481_1494del	p.(K494Ifs*12)	Exon 15	CD080877
c.1516+1G>A	p.(?)	Intron 15	CS152761
c.1516+1G>C	p.(I482Gfs*10)	Intron 15	CS080721
c.1516+3A>G	p.(I482Gfs*10)	Intron 15	LOVD-ID:OPA1_000582
c.1560_1562del	p.(E521del)	Exon 16	CD094188
c.1645dup	p.(S549Ffs*13)	Exon 17	CI002739
c.1652_1654del	p.(C551del)	Exon 17	CD012272
c.1687C>T	p.(Q563*)	Exon 17	CM126970
c.1705+1G>T	p.(V556Qfs*40)	Intron 17	CS061312
c.1771-2A>G	p.(?)	Intron 18	CS094209
c.1778T>C	p.(L593P)	Exon 19	CM094166
c.1847+4_1847+7del	p.(?)	Intron 19	CD094193
c.1879A>T	p.(R627*)	Exon 20	CM094169
c.1881_1882del	p.(R627Sfs*7)	Exon 20	CD031533
c.1892_1893del	p.(H631Rfs*3)	Exon 20	CD094194
c.2013G>A	p.(A673Rfs*3)	Exon 20	CS061314
c.2014-1G>A	p.(V672Lfs*14)	Intron 20	CS061313
c.2119G>T	p.(E707*)	Exon 21	CM130780
c.2125_2138del14ins12	p.(I709Gfs*7)	Exon 21	CX012305
c.2131C>T	p.(R711*)	Exon 21	CM013590
c.2142G>A	p.(W714*)	Exon 21	CM1410789
c.2197C>T	p.(R733*)	Exon 22	CM086329
c.2241del	p.(F747Lfs*53)	Exon 22	LOVD-ID:OPA1_000579
c.2354A>G	p.(Q785R) and p.(T759Mfs*5)	Exon 23	CM012175
c.2355+1G>A	p.(T759Mfs*5)	Intron 23	CS067103
c.2396T>A	p.(L799*)	Exon 24	CM080472
c.2470C>T	p.(R824*)	Exon 24	CM066156
c.2569C>T	p.(R857*)	Exon 25	CM094173
c.2586_2587insA	p.(Y863Ifs*9)	Exon 25	CI080973
c.2613+1G>C	p.(?)	Intron 25	CS012219
c.2614-1G>A	p.(?)	Intron 25	CS094217
c.2614-9A>G	p.(L872_Q884del)	Intron 25	CS068103
c.2707+2T>C	p.(E873Gfs*3)	Intron 26	CS012220
c.2708-1G>T	p.(V903Gfs*3)	Intron 26	CS061315
c.2708_2711del	p.(V903Gfs*3)	Exon 27	CD002708
c.2713C>T	p.(R905*)	Exon 27	CM012176
c.2729T>A	p.(V910D)	Exon 27	CM080468
c.2790_2798delins9	p.(R932_V933delinsHR)	Exon 27	CX080992
c.2797G>A	p.(V933I)	Exon 27	CM104765
c.2815del	p.(L939Sfs*29)	Exon 27	CD104766
c.2818+1G>A	p.(?)	Intron 27	CS170561
c.2818+5G>A	p.(V903_K940delinsE)	Intron 27	CS080722
c.2819-2A>C	p.(K940_V942delinsI)	Intron 27	CS080723
c.2825_2828del	p.(V942Efs*25)	Exon 28	CD002709
c.2844dup	p.(L949Tfs*2)	Exon 28	CI1814095
g.193,310,511_193,312,933 delins193,310,603_193,311,825 [193,310,603_193,310,540inv]	p.(0?)	Exon 1	PMID:33243194
c.(?_-1)_(2818+1_2819–1)del	p.(0?)	Exon 1–27	CG091336
c.(?_-1)_(624+1_625–1)del	p.(0?)	Exon 1–5	CG091338
c.871-162_985-1789delinsGTGATTGATGCA	p.(?)	Exon 9	CG091339
c.2356-586_2497-616del	p.(C786_L832del)	Exon 24	CG091337
c.(2496+1_2497–1)_(2707+1_2708–1)del	p.(I833Lfs*4)	Exon 25–26	CG112206
c.678+674_984+2026dup	p.(L227_K328dup)	Exon 7–9	CN091340
c.(?_-1)_(*3211_?)del	p.(0?)	Entire gene	CG091335

^a^protein level for splicing variant was given when established by cDNA analysis

^b^in case variant is not listed in HGMD, identifier of LOVD or Pubmed is given

**Table 3:** Novel variants identified in this study.

NM_015560.2^a^	NP_056375.2^b^	Exon/Intron	Alleles in gnomAD	ACMG/AMP criteria^c^	ACMG/AMP classification
c.50del	p.(L17*)	Exon 2	-	PVS1;PM2	Likely pathogenic (I)
c.86del	p.(P29Hfs*20)	Exon 2	-	PVS1;PM2	Likely pathogenic (I)
c.295_301dup	p.(R101Qfs*22)	Exon 2	-	PVS1;PM2	Likely pathogenic (I)
c.394del	p.(Y132Ifs*32)	Exon 3	-	PVS1;PM2;PP1	Pathogenic (Ia)
c.557-8_557-3del	p.(?)	Intron 4	-	PM2;PP1	Likely pathogenic (II)
c.572C>T	p.(T191M)	Exon 5	1/152120	PP3	VUS
c.586dup	p.(T196Nfs*6)	Exon 5	-	PVS1;PM2	Likely pathogenic (I)
c.814C>T	p.(L272F)	Exon 8	-	PM1;PM2;PM5;PP1;PP3	Pathogenic (IIIa)
c.838_839insT	p.(A280Vfs*4)	Exon 8	-	PVS1;PM2	Likely pathogenic (I)
c.874G>T	p.(V292F)	Exon 9	-	PM1;PM2;PP3	VUS
c.975del	p.(P326Qfs*4)	Exon 9	-	PVS1;PM2	Likely pathogenic (I)
c.989_994del	p.(T330_L331del)	Exon 10	-	PM1;PM2;PM4	Likely pathogenic (IV)
c.992dup	p.(S332Efs*2)	Exon 10	-	PVS1;PM2	Likely pathogenic (I)
c.1065+6T>C	p.(V329_D355del)	Intron 10	-	PM2;PS3	Likely pathogenic (II)
c.1140_1140+1insT	p.(?)	Intron 11	-	PVS1;PM2	Likely pathogenic (I)
c.1154_1161del	p.(N385Rfs*11)	Exon 12	-	PVS1;PM2	Likely pathogenic (I)
c.1180A>G	p.(M394V)	Exon 12	4/152182	PP3	VUS
c.1212+1G>C	p.(?)	Intron 12	-	PVS1;PM2;PP1	Pathogenic (Ia)
c.1212+4del	p.(T381_N404del)	Intron 12	-	PM2;PS3	Likely pathogenic (II)
c.1213-1G>A	p.(?)	Intron 12	-	PVS1;PM2	Likely pathogenic (I)
c.1282A>G	p.(N428D)	Exon 13	-	PM1;PM2;PP1;PP3	Likely Pathogenic (II)
c.1360C>T	p.(Q454*)	Exon 14	-	PVS1;PM2	Likely pathogenic (I)
c.1558G>T	p.(E520*)	Exon 16	-	PVS1;PM2	Likely pathogenic (I)
c.1632_1638del	p.(S545Qfs*62)	Exon 17	-	PVS1;PM2;PP1	Pathogenic (Ia)
c.1723G>A	p.(E575K)	Exon 18	-	PM2;PP3	VUS
c.1734G>A	p.(W578*)	Exon 18	-	PVS1;PM2	Likely pathogenic (I)
c.1754del	p.(L585Rfs*24)	Exon 18	-	PVS1;PM2;PP1	Pathogenic (Ia)
c.1770+2T>G	p.(?)	Intron 18	-	PVS1;PM2	Likely pathogenic (I)
c.1864C>T	p.(Q622*)	Exon 20	-	PVS1;PM2	Likely pathogenic (I)
c.2013G>C	p.(?)	Exon 20	-	PS1;PM2;PP1;PP3	Pathogenic (II)
c.2014-10A>G	p.(V672*)	Intron 20	-	PM2;PS3	Likely pathogenic (II)
c.2032C>T	p.(Q678*)	Exon 21	-	PVS1;PM2	Likely pathogenic (I)
c.2103delinsTAAG	p.(L700_K701insN)	Exon 21	-	PM2;PM4;PP3	VUS
c.2150_2151del	p.(F717Cfs*20)	Exon 21	-	PVS1;PM2;PP1	Pathogenic (Ia)
c.2237_2240del	p.(Y746Lfs*53)	Exon 22	-	PVS1;PM2	Likely pathogenic (I)
c.2267T>C	p.(L756P)	Exon 22	-	PM2;PP1;PP3	Likely pathogenic (II)
c.2347dup	p.(Q783Pfs*8)	Exon 23	-	PVS1;PM2	Likely pathogenic (I)
c.2356-8T>G	p.(C786Ffs*7)	Intron 23	-	PM2;PS3	Likely pathogenic (II)
c.2511G>A	p.(W837*)	Exon 25	-	PVS1;PM2	Likely pathogenic (I)
c.2540_2564dup	p.(C856Nfs*7)	Exon 25	-	PVS1;PM2	Likely pathogenic (I)
c.2585dup	p.(Y862*)	Exon 25	-	PM2;PVS1	Likely pathogenic (I)
c.2704G>T	p.(E902*)	Exon 26	-	PVS1;PM2	Likely pathogenic (I)
c.2795_2801delinsTC	p.(R932Lfs*10)	Exon 27	-	PVS1;PM2;PP1	Pathogenic (Ia)
c.(1847+1_1848–1)_(2013+1_2014–1)del	p.(E617Lfs*14)	Exon 20	-	PVS1;PM2	Likely pathogenic (I)
c.(1847+1_1848–1)_(*3211_?)del	p.(0?)	Exon 20–28	-	PVS1;PM2	Likely pathogenic (I)
c.(2013+1_2014–1)_(2355+1_2356–1)del	p.(V672_Q785del)	Exon 21–23	-	PVS1;PM2	Likely pathogenic (I)
c.(2707+1_2708–1)_(2818+1_2819–1)del	p.(V903Efs)	Exon 27	-	PVS1;PM2;PP1	Pathogenic (Ia)
c.(448+1_449–1)_(2355+1_2356–1)del	p.(E150Vfs*5)	Exon 4–23	-	PVS1;PM2	Likely pathogenic (I)

^a^breakpoints have not been defined for CNVs

^b^protein level for splicing variant was given when established by cDNA analysis

^c^categories published in the ACMG/AMP guidelines [86]. VUS, variant of uncertain significance.

The 156 unique variants identified in our cohort spread along the whole gene and include 98 variants located in exons and 44 variants located in introns. One variant, a 38 bp deletion, affects both exonic and intronic sequence. In addition, we have identified 13 structural variants: 12 are copy number variants (CNVs) comprising one or more exons, and one is an inversion that comprises exon 1. Exonic variations include 55 single nucleotide substitutions (25 of them generating a stop codon, three of them possibly affecting splicing, and 27 of them causing an amino acid substitution), and 27 deletions of one to few nucleotides (one of them generating a stop codon, 22 causing a frameshift, and four causing an in frame deletion). Furthermore, exonic variants comprise eleven duplications or insertions of one or few nucleotides (one of them generating a stop codon, and ten causing a frameshift) and five insertion/deletion variants (two causing a frameshift, two causing an in frame insertion/deletion, and one causing a single amino acid substitution). Of the 44 intronic variations, 37 are single nucleotide substitutions. Twenty-three of them affect the highly conserved GT and AG splice acceptor and splice donor dinucleotides. In the following, these variants are referred to as canonical splice site (CSS) variants. Twelve variants are located in the vicinity but outside the highly conserved splice acceptor and donor sites. In the following, these variants are referred to as non-canonical splice site (NCSS) variants. Two variants are located deep in an intron. Five intronic variants are deletions of one to few nucleotides (three affecting CSSs, and two NCSSs), and two are duplications or insertions of one nucleotide (both affecting CSSs).

When grouping all 156 unique variants according to their deduced effect on protein function, 34 variants are predicted to alter single amino acid residues (27 missense and 7 in frame variants, shown as pink and green dots in Fig 1, respectively), 74 variants are predicted to result in a truncated protein (25 nonsense, 36 frameshift, and 13 structural variants, shown as blue and black dots and horizontal lines in Fig 1, respectively) and 48 variants (putatively) affect splicing (shown as grey dots in Fig 1). Twenty-seven of the splicing variants have been validated by us either by analyzing cDNA derived from patients´ blood samples or by heterologous splice assays using minigenes [58, 76, 90] (results for yet unpublished NCSS variants are shown in S1 and S2 Figs). The two deep intronic variants act by the insertion of pseudoexons, thereby leading to a frameshift and premature termination codon (PTC) in the aberrant transcripts. Fifteen splicing variants were shown to lead to the skipping of the respective exon, with four of them leading to a frameshift and PTC while eleven retain the reading frame. Ten variants were shown to activate exonic or intronic cryptic splice sites, thereby leading to a PTC in eight cases and causing an in frame deletion in two cases. The infered effect on protein level of these experimentally validated splicing variants is shown in Tables 2 and 3.

Assuming that all splice variants and structural variants give rise to transcripts that either harbor a PTC or lack important protein domains, 78% of the variants in our cohort can be considered *null* alleles. Most of the 27 missense variants cluster to the GTPase domain encoded by exons 9–16 (see Fig 1), which is essential for protein function. Several missense variants in this region have been demonstrated to cause a severe loss of mitochondrial fusion activity [91, 92].

Several variants were recurrent. Table 4 lists the ten most frequent alleles found in our cohort. The most frequent variant, c.2708_2711del/p.(V903Gfs*3), was found in 39 of 278 families, accounting for 14% of disease-causing alleles.

**Table 4:** Ten most frequent variants in our cohort.

Variant	Allele count
c.2708_2711del/p.(V903Gfs*3)	39
c.869G>A/p.(R290Q)	8
c.870+5G>A/p.(?)	8
c.2569C>T/p.(R857*)	7
c.1313A>T/p.(D438V)	6
c.635_636del/p.(K212Rfs*4)	5
c.1212+1G>A/p.T381_N404del)	5
c.2241del/p.(F747Lfs*53)	5
c.154C>T/p.(R52*)	4
c.1096C>T/p.(R366*)	4

Five index patients harbored two variants each in the *OPA1* gene. In three of them, the two respective variants were shown to be *in trans* configuration by family segregation analysis. Two of these patients carried a splice variant on one allele and the p.(I382M) missense variant on the counter allele. The latter is considered a hypomorphic allele that causes a severe DOAplus phenotype when occurring *in trans* with a *null* allele [58, 67]. Indeed, both patients were diagnosed with a Behr syndrome-like phenotype [58, 69]. Another patient was shown to be compound heterozygous for the two missense variants p.(E270K) and p.(R290W). This patient presented with a much more severe phenotype than her single heterozygous parents and siblings, indicating that these two *OPA1* alleles behave semi-dominantly [18]. Zygosity (i.e. *cis* or *trans* configuration of two variants in heterozygous state) could not be established in another two patients harboring two variants: one of them carried a novel nonsense p.(E520*) and a novel missense variant p.(M394V), while the other carried a known in frame insertion/deletion variant p.(I313E) and a known missense variant p.(T376A).

**Fig 2:**
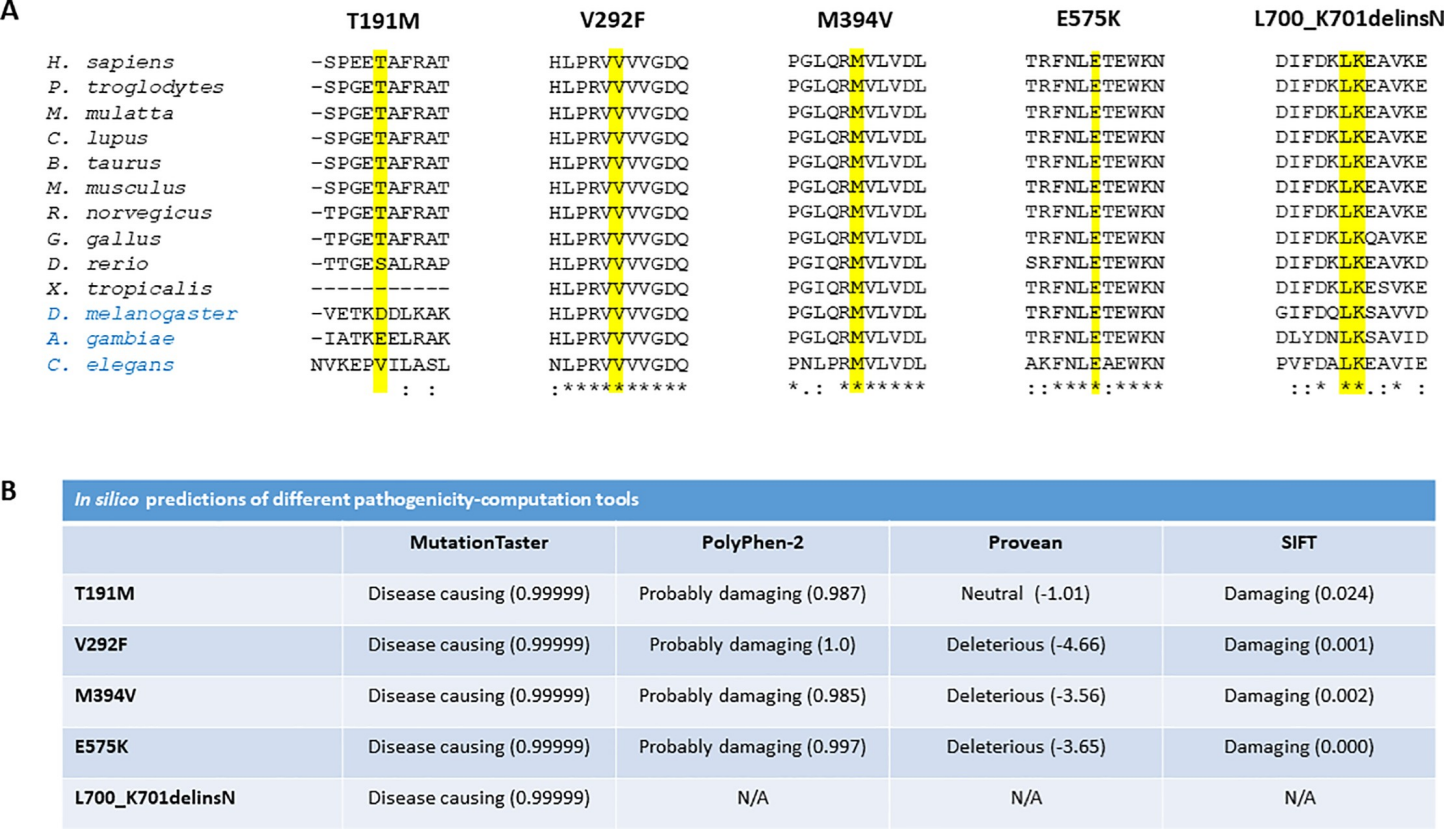
Novel amino acid substitution variants in *OPA1* classified as VUS. A) Multiple sequence alignment of *Homo sapiens* OPA1 against its orthologues from twelve other species was performed using Clustal Omega (https://www.ebi.ac.uk/Tools/msa/clustalo/). The mutated amino acid residues 191, 292, 394, 575, and 700–701 are highlighted in yellow. Reference sequences were taken from NCBI and are as follows: *H*. *sapiens* (NP_570849.2), *P*. *troglodytes* (XP_003310225.1), *M*. *mulatta* (XP_001087037.2), *C*. *lupus* (XP_005634679.1), *B*. *taurus* (NP_001179890.1), *M*. *musculus* (NP_001186106.1), *R*. *norvegicus* (NP_598269.3), *G*. *gallus* (NP_001034398.1), *D*. *rerio* (NP_001007299.1), *X*.*tropicalis* (NP_001120510.1), *D*. *melanogaster* (NP_725369.1), *A*. *gambiae* (XP_309360.3), and *C*. *elegans* (NP_495986.3). Vertebrates are labeled in black; non-chordates are labeled in blue. B) *In silico* predictions of different pathogenicity-computation tools. N/A, not applicable.

Of the 156 unique variants identified by us, 48 were neither listed in HGMD and the LOVD database for *OPA1* (as of April 2021), nor have they been published in a journal searchable on Pubmed (as of April 2021). We followed the ACMG/AMP guidelines to classify these novel variants. Assignment of individual criteria and final classification is given in Table 3. Since haploinsufficiency has been proposed as the predominant disease mechanism for *OPA1* variants [18, 57], the criterion PVS1 was used for nonsense, frameshift, and CSS variants as well as for structural variants. Variants located in the GTPase domain of the OPA1 protein were assigned the PM1 criterion. All novel variants but two fulfilled criterion PM2, since no allele frequency was reported in gnomAD. The criterion PS3 was used for variants that have been analyzed on cDNA level. For novel missense variants at an amino acid residue where a different missense change determined to be pathogenic has been reported in prior studies, criterion PM5 was used. The criterion PP3 was used for missense variants that were classified as “disease causing” by MutationTaster which predicts pathogenicity by a combination of criteria, including the conservation on nucleotide and amino acid level, potential loss of functional protein domains, and the effect on splicing [79]. Variants that were found to segregate within families were assigned the criterion PP1. Following the ACMG/AMP guidelines, nine variants were classified as pathogenic, 34 as likely pathogenic and five as variant of uncertain significance (VUS). Among the variants classified as VUS are four missense variants, and one in frame insertion/deletion variant. To provide additional information for these variants, we performed an amino acid alignment of the OPA1 protein in different species (Fig 2A). The respective amino acid residues are fully conserved for variants p.(V292F), p.(M394V), p.(E575K) and p.(L700_K701delinsN), whereas the threonine residue of variant p.(T191M) is conserved in mammals and birds only. Accordingly, four different *in silico* bioinformatic tools predicted the variants to be pathogenic with high agreement (Fig 2B).

## Discussion

The prevalence of putatively pathogenic *OPA1* variants in our cohort is 36.8% (278/755). This value is at the lower end of the range of 32–90% observed in other studies [18, 23]. There are two possible explanations. First of all, our cohort is not homogeneous with respect to clinical phenotyping. The patients in our study were diagnosed and recruited at different centers throughout Germany and Europe. The diagnosis of DOA was based on a history of gradual, bilateral vision loss associated with the presence of central or caecocentral scotoma and symmetric temporal or diffuse optic disc pallor. However, clinical records were not available for all patients. In addition, considering the known reduced penetrance and the presence of asymptomatic carriers in DOA [6, 19, 74, 75], the cohort includes cases in which an autosomal dominant mode of inheritance could not be unequivocally established. Eventually, not all of the patients in our cohort might be DOA cases, especially when considering the overlapping phenotypes seen within the group of optic neuropathies [2]. Of note, in our cohort 429 cases are male while 311 are female (see Table 1). A gender bias (i.e., a higher incidence in men) is not typical for DOA, but for LHON [93]. It is tempting to speculate that the observed gender bias in our cohort is caused by a large proportion of LHON cases that were misdiagnosed as DOA. However, as the bias is still present when considering only those cases harboring pathogenic variants in *OPA1* (151 male subjects, 115 female subjects), the larger proportion of male cases must be caused by other factors about which we could only speculate at the moment.

DOA as well as recessively inherited optic neuropathies are genetically heterogeneous. Accordingly, we found disease-causing variants in *OPA3* [31], *SSBP1* [41], *ACO2* [46], and *TMEM126A* [94] in some patients. In addition, individual cases harbored pathogenic variants in *WFS1*, *MFN2*, and *DNAJC30* (unpublished), as well as in the mitochondrial DNA [95]. The diagnosis in the latter patients was changed from DOA to LHON. The diagnosis in those patients harboring pathogenic variants in genes exclusively asscociated with recessively inherited optic neuropathies was changed accordingly. In total, 5.5% of cases in our cohort could be solved by pathogenic variants in genes other than *OPA1*. Note that we cannot give actual prevalences since the abovementioned genes have not been analysed in the entire cohort. The second possible reason for the relatively low prevalence of patients harboring *OPA1* variants in our cohort might be due to the fact that our genetic testing during the early 2000s was based on less sensitive methods like SSCP and DHPLC. A comprehensive re-analysis of all unsolved patients in our cohort has not been performed. In addition, even with NGS platforms, which are used for genetic diagnostic testing of newly recruited patients since 2012, structural variants as well as variants in non-coding regions remain challenging to identify and interpret.

Information on family history was available for roughly two-third of patients in our cohort that harbored *OPA1* variants. While 38 patients (13.6%) reported a negative family history, 139 (49.8%) reported one or more affected family members. Genotyping of available family members confirmed the presence of multiple affected subjects in 100 families. In contrast, only 25.3% of unsolved patients in our cohort reported affected family members while 24.1% reported a negative family history. The higher rate of simplex cases among unsolved cases is in line with the observation that multi-generation families are more likely to harbor disease-causing variants in the *OPA1* gene [18].

A limitation of our study is the lack of detailed clinical data in a considerable number of patients which hampers the assessment of phenotype-genotype correlations. Several studies have indicated that missense variants in *OPA1* tend to be associated with more severe phenotypes [12, 13, 15, 96]. We can neither confirm nor deny such a correlation due to the lack of comprehensive clinical data in our study. However, in line with other studies, we observed more severe phenotypes in the few biallelic *OPA1* patients [18, 58].

With 39 index cases, the c.2708_2711del/p.(V903Gfs*3) variant is the most frequent *OPA1* disease-associated allele in our cohort. Since our cohort mainly comprises patients of German origin, we found the p.(V903Gfs*3) variant mainly in German patients (n = 27), but also in patients originating from France (n = 4), Italy (n = 4), Belgium (n = 2) and the Middle East (n = 2). The c.2708_2711del/p.(V903Gfs*3) variant has been described repeatedly in the literature, including studies performed in Italy, France, Denmark, UK, China and the USA [17, 68, 71, 97–104]. This suggests a mutation hotspot rather than a founder effect. Another mutation hotspot seems to be the splice donor site of exon 12, which we found to be altered in 13 index cases.

Of note, of the 46 intronic variants that are located at the canonical splice sites or in their vicinity, 14 affect the acceptor site, and 32 affect the donor site, accounting for a ratio of 2:1 of donor to acceptor splice site mutations. The reason for this imbalance is unknown but a similar value has been reported in a study that investigated 478 splice mutations in 38 different human genes [105].

Among the 156 unique variants identified in this study, 48 (30.8%) have not been reported before. This demonstrates that, although the identification of the *OPA1* gene as the underlying cause of DOA dates back 20 years [16, 17], the mutation spectrum of this gene is still far from being saturated. We have applied the ACMG/AMP guidelines to classify all novel variants identified in our study. After having validated that five as yet unpublished NCSS variants exerted a splicing defect (see S1 and S2 Figs), 43 of the 48 novel variants could be classified either as pathogenic or likely pathogenic. Five novel amino acid substitution variants had to be classified as VUS. Novel missense variants will always be classified as VUS without family segregation data or functional analysis supporting their pathogenicity. Naturally, this “gray zone category” is equally unsatisfying for geneticists, physicians and patients. A limitation of our study is that we could not evaluate the pathogenicity of the five novel amino acid substitution variants. One approach to assess the impact of a variant on OPA1 protein function is to measure the oxygen consumption rates of urine cells [106]. Other studies have successfully applied targeted metabolomics to explore the different signatures of *OPA1* variants expressed in Opa1 deleted mouse embryonic fibroblasts [107, 108]. These kind of investigations were beyond the scope of the present study. Instead, we performed an *in silico* analysis using different bioinformatic tools (Fig 2B). Concordant results were obtained for missense variants V292F, M394V, and E575K, which were predicted to be disease-causing by four algorithms. Variant T191M was predicted to be pathogenic by three algorithms. Variant L700_K701delinsN could only be assessed with one algorithm and was predicted to be pathogenic. Of note, two of the novel missense variants affect fully conserved amino acid residues in the GTPase domain, where numerous pathogenic missense variants have been reported to date (see Figs 1 and 2). Probably no one would doubt the pathogenic effect of these variants. However, according to the ACMG/AMP guidelines, the classification of these variants has to be VUS until additional evidence is supporting their pathogenicity.

Not only missense variants, but also novel NCSS variants have to be classified as VUS without further supporting evidence. Among the 48 splicing variants we have identified, 21 are outside the highly conserved GT and AG splice acceptor and splice donor dinucleotides. For 13 of them, we performed further analyses on cDNA level (either by direct mRNA analysis or by minigene assays if RNA from patients was not available) and could confirm that they cause a splicing defect [58, 76, 90] (details of splicing analyses of yet unpublished variants are shown in S1 and S2 Figs). According to the ACMG/AMP guidelines, we were able to upgrade the classification of these variants from VUS to likely pathogenic. This demonstrates that variants classified as VUS have the true potential to be disease-causing and should always be included in the diagnostic reports. On the other hand, functional analysis of individual variants can hardly be implemented in routine workflows of diagnostic laboratories. Hence, the validation of VUS variants is largely dependent on research-based efforts.

## Supporting information

S1 Fig Direct cDNA analysis from patients´ blood samples. Following reverse transcription, the region of interest was amplified from cDNA. Note that RT-PCR products were sequenced without prior subcloning. The sequence electropherograms of variants c.1065+6T>C (A), c.1212+4del (B), and c.1516+3A>G (C) show an overlay of wildtype (black letters) and mutant sequences (grey letters), starting at the respective exon-exon junction and indicative of exon skipping. While the skipping of exon 10 and exon 12 is not predicted to change the reading frame, the skipping of exon 15 is predicted to lead to a frameshift and PTC. (D-F) Quantitative analysis of fluorescently labeled RT-PCR products. The fragment size scale is given on the x-axis and the fluorescence intensity (in arbitrary units) is given on the y-axis. Relative amounts of each fragment are given for the corresponding peak as determined by Gene Mapper. In each graph, the larger product corresponds to the correctly spliced transcript while the smaller product is the aberrant transcript with exon skipping. Wildtype and mutant allele were found to be expressed in approximately equal amounts in the two patients heterozygous either for variant c.1065+6T>C or c.1212+4del. In contrast, the mutant transcript is clearly less abundant in the patient heterozygous for variant c.1516+3A>G, most probably due to NMD.
(TIF)

S2 Fig *In vitro* splice assays of variants c.2014-10A>G (A+C) and c.2356-8T>G (B+D). (A+B) Agarose gel electrophoresis of RT-PCR products. Gel loading is as follows: A size standard (low molecular weight DNA ladder, NEB) is loaded in the leftmost lane. The RT-PCR product derived from HEK293T cells transfected with the wildtype minigene construct is shown in lane 2, while the RT-PCR product obtained upon transfection with the mutant minigene construct is shown in lane 3. RT-PCRs from transfection with empty pSPL3 vector (lane 4) and untransfected HEK293T cells (lane 5) served as controls. NRT (lane 6), no reverse transcriptase control; NTC (lane 7), no template control. Schemes of the amplified products are presented next to the agarose gel image (not drawn to scale). Blue boxes represent pSPL3 resident exons tat1 and tat2, and pink boxes *OPA1* exons, respectively. The dotted edging represents retained intronic sequence. The green arrows indicate the location of the RT-PCR primers. (C+D) Sequencing analysis of RT-PCR products. Only the relevant junctions are shown. The minigene splicing assay performed for variant c.2014-10A>G (C) showed that the last nine nucleotides of intron 20 (given in lowercase letters) are spliced between the resident pSPL3 exon tat1 and exon 21 of the *OPA1* gene. The aberrant transcript would lead, if translated, to the generation of a PTC (p.(V672*)). The minigene splicing assay performed for variant c.2356-8T>G (D) showed that the last seven nucleotides of intron 24 (given in lowercase letters) are spliced between the resident pSPL3 exon tat1 and exon 24 of the *OPA1* gene. The aberrant transcript would lead, if translated, to the insertion of six altered amino acid residues followed by a PTC (p.(C786Ffs*7)).
(TIF)

S1 Raw images (PDF)
