# Supplementary figures and images for: Mutation spectrum of the OPA1 gene in a large cohort of patients with suspected dominant optic atrophy: Identification and classification of 48 novel variants
Source: PLoS One. 2021 Jul 9;16(7):e0253987. doi: 10.1371/journal.pone.0253987 (PMC8270428; doi:10.1371/journal.pone.0253987)

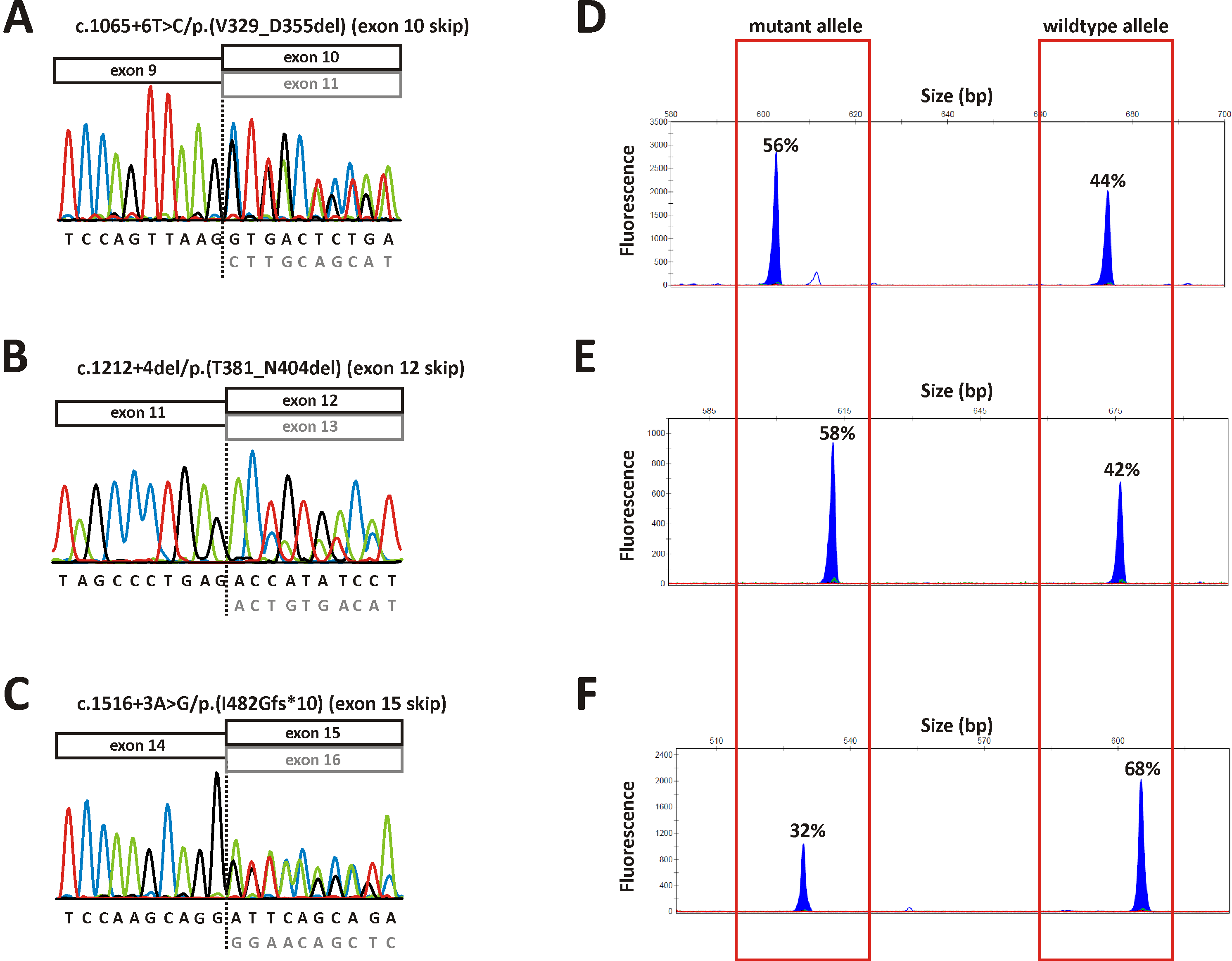

Supplement: S1 Fig — Following reverse transcription, the region of interest was amplified from cDNA. Note that RT-PCR products were sequenced without prior subcloning. The sequence electropherograms of variants c.1065+6T>C (A), c.1212+4del (B), and c.1516+3A>G (C) show an overlay of wildtype (black letters) and mutant sequences (grey letters), starting at the respective exon-exon junction and indicative of exon skipping. While the skipping of exon 10 and exon 12 is not predicted to change the reading frame, the skipping of exon 15 is predicted to lead to a frameshift and PTC. (D-F) Quantitative analysis of fluorescently labeled RT-PCR products. The fragment size scale is given on the x-axis and the fluorescence intensity (in arbitrary units) is given on the y-axis. Relative amounts of each fragment are given for the corresponding peak as determined by Gene Mapper. In each graph, the larger product corresponds to the correctly spliced transcript while the smaller product is the aberrant transcript with exon skipping. Wildtype and mutant allele were found to be expressed in approximately equal amounts in the two patients heterozygous either for variant c.1065+6T>C or c.1212+4del. In contrast, the mutant transcript is clearly less abundant in the patient heterozygous for variant c.1516+3A>G, most probably due to NMD. (TIF) [file pone.0253987.s001.TIF]

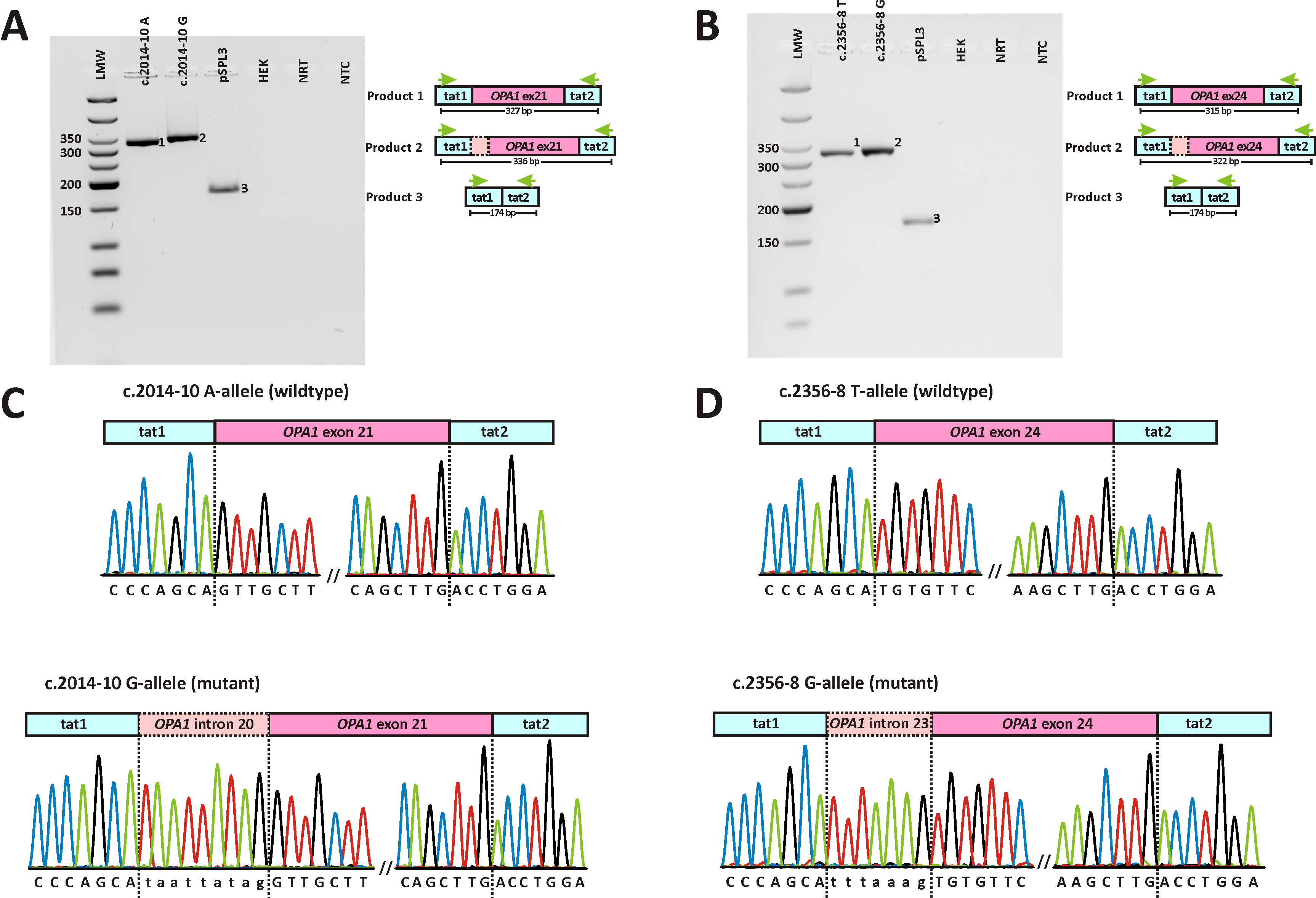

Supplement: S2 Fig — (A+B) Agarose gel electrophoresis of RT-PCR products. Gel loading is as follows: A size standard (low molecular weight DNA ladder, NEB) is loaded in the leftmost lane. The RT-PCR product derived from HEK293T cells transfected with the wildtype minigene construct is shown in lane 2, while the RT-PCR product obtained upon transfection with the mutant minigene construct is shown in lane 3. RT-PCRs from transfection with empty pSPL3 vector (lane 4) and untransfected HEK293T cells (lane 5) served as controls. NRT (lane 6), no reverse transcriptase control; NTC (lane 7), no template control. Schemes of the amplified products are presented next to the agarose gel image (not drawn to scale). Blue boxes represent pSPL3 resident exons tat1 and tat2, and pink boxes OPA1 exons, respectively. The dotted edging represents retained intronic sequence. The green arrows indicate the location of the RT-PCR primers. (C+D) Sequencing analysis of RT-PCR products. Only the relevant junctions are shown. The minigene splicing assay performed for variant c.2014-10A>G (C) showed that the last nine nucleotides of intron 20 (given in lowercase letters) are spliced between the resident pSPL3 exon tat1 and exon 21 of the OPA1 gene. The aberrant transcript would lead, if translated, to the generation of a PTC (p.(V672*)). The minigene splicing assay performed for variant c.2356-8T>G (D) showed that the last seven nucleotides of intron 24 (given in lowercase letters) are spliced between the resident pSPL3 exon tat1 and exon 24 of the OPA1 gene. The aberrant transcript would lead, if translated, to the insertion of six altered amino acid residues followed by a PTC (p.(C786Ffs*7)). (TIF) [file pone.0253987.s002.TIF]

Suppl FigS2A

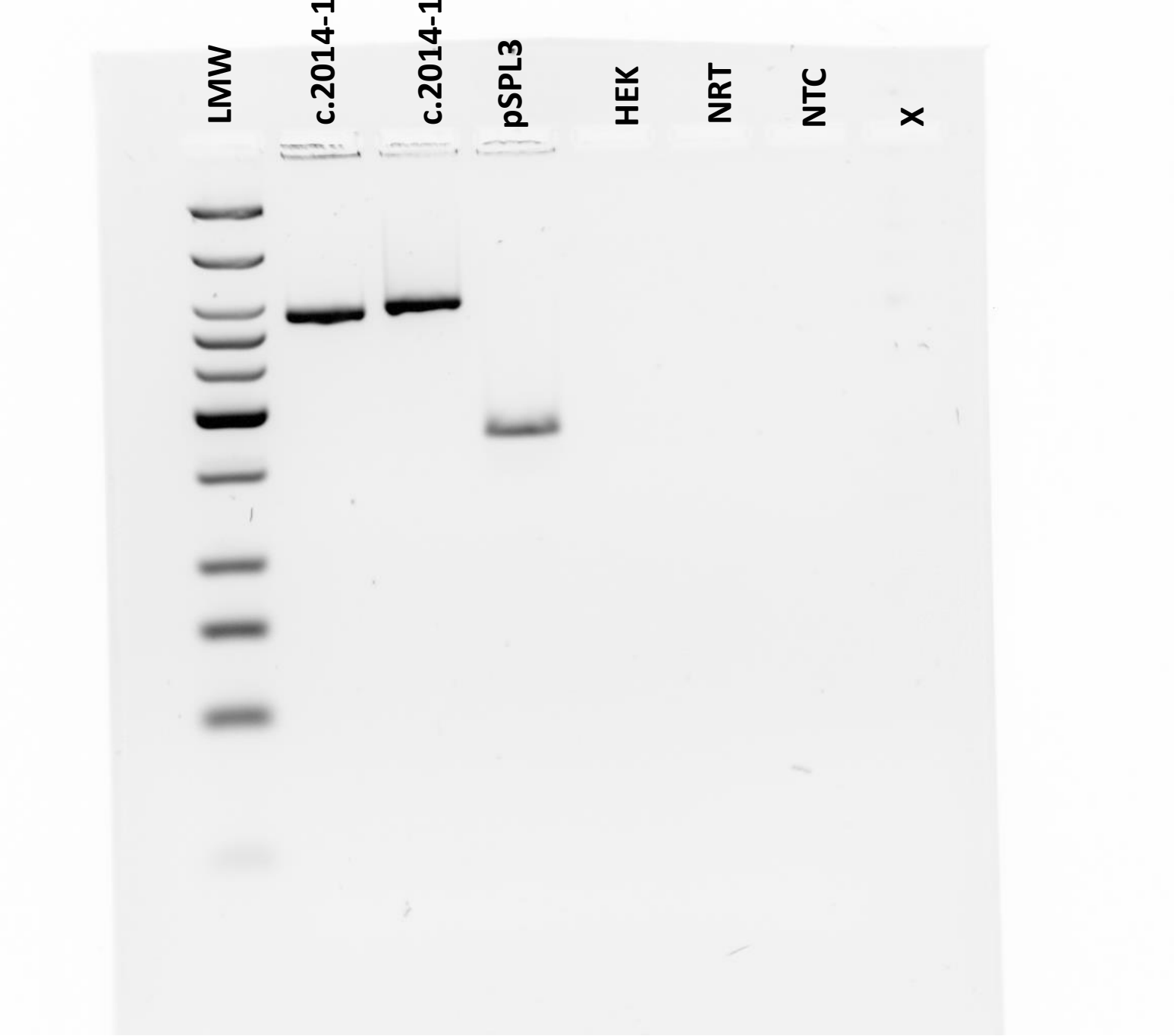

Suppl FigS2B

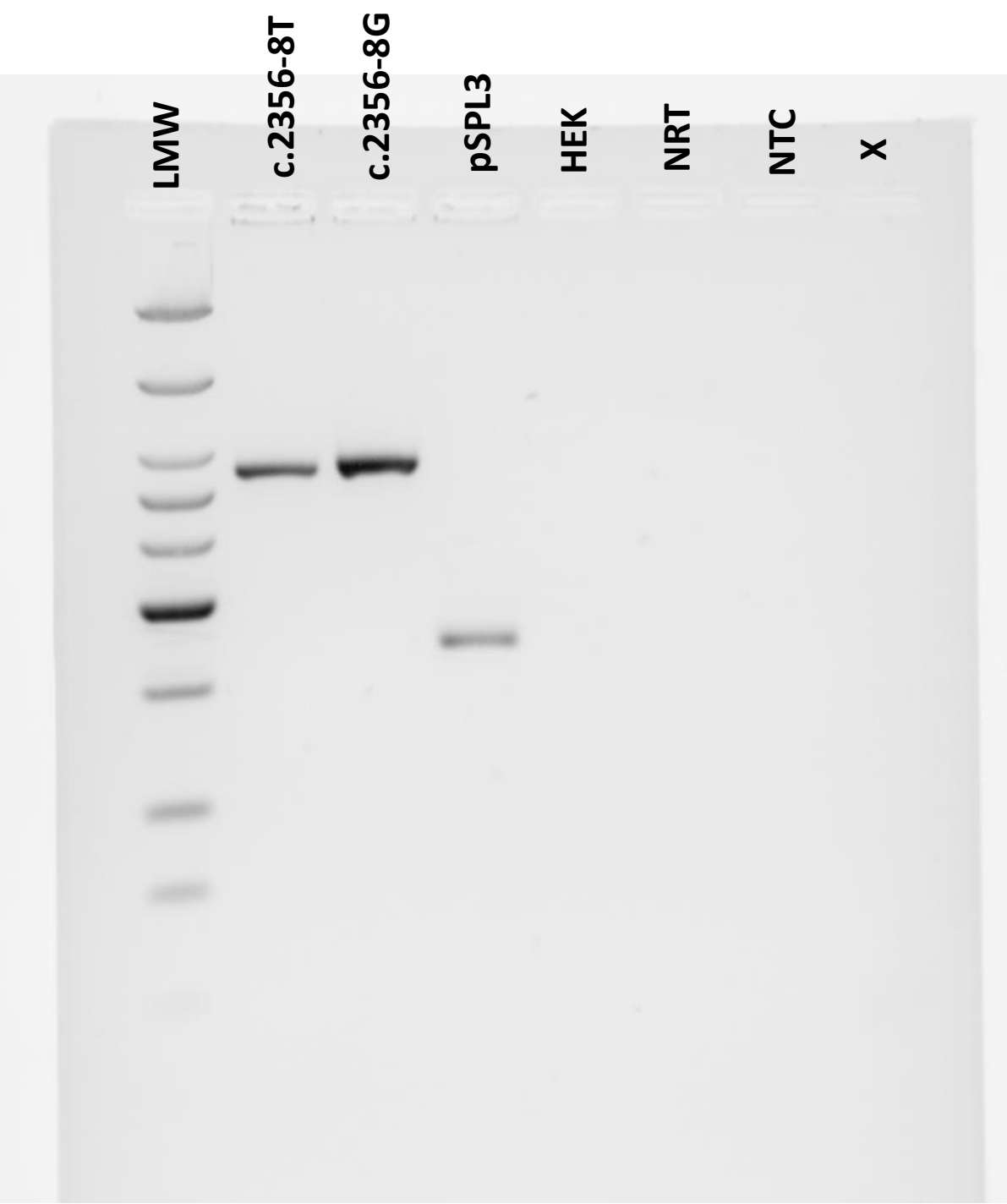

Supplement: S1 Raw images — (PDF) [file pone.0253987.s003.pdf]
